# Novel Insights into YB-1 Signaling and Cell Death Decisions

**DOI:** 10.3390/cancers13133306

**Published:** 2021-07-01

**Authors:** Aneri Shah, Jonathan A. Lindquist, Lars Rosendahl, Ingo Schmitz, Peter R. Mertens

**Affiliations:** 1 Clinic of Nephrology and Hypertension, Diabetes and Endocrinology, Otto-von-Guericke University, 39120 Magdeburg, Germany; aneri.shah@ovgu.de (A.S.); lars.rosendahl@st.ovgu.de (L.R.); peter.mertens@med.ovgu.de (P.R.M.); 2 Department of Molecular Immunology, ZKF2, Ruhr-University Bochum, 44801 Bochum, Germany; ingo.schmitz@ruhr-uni-bochum.de

**Keywords:** YB-1, TNF, NF-κB, apoptosis

## Abstract

**Simple Summary:**

Signals that determine cell survival or death are essential for maintaining tissue homeostasis. Cell death promotes the removal of unwanted cells; however, a failure of cells to die or cells dying when they should not can exacerbate inflammation, and the former is a causative factor in cancerous diseases. YB-1 plays critical roles in cell proliferation and differentiation, stress responses, and tumorigenesis. In this review, we have summarized recent insights into the role of YB-1 in signaling cell survival and apoptosis.

**Abstract:**

YB-1 belongs to the evolutionarily conserved cold-shock domain protein family of RNA binding proteins. YB-1 is a well-known transcriptional and translational regulator, involved in cell cycle progression, DNA damage repair, RNA splicing, and stress responses. Cell stress occurs in many forms, e.g., radiation, hyperthermia, lipopolysaccharide (LPS) produced by bacteria, and interferons released in response to viral infection. Binding of the latter factors to their receptors induces kinase activation, which results in the phosphorylation of YB-1. These pathways also activate the nuclear factor kappa-light-chain-enhancer of activated B cells (NF-κB), a well-known transcription factor. NF-κB is upregulated following cellular stress and orchestrates inflammatory responses, cell proliferation, and differentiation. Inflammation and cancer are known to share common mechanisms, such as the recruitment of infiltrating macrophages and development of an inflammatory microenvironment. Several recent papers elaborate the role of YB-1 in activating NF-κB and signaling cell survival. Depleting YB-1 may tip the balance from survival to enhanced apoptosis. Therefore, strategies that target YB-1 might be a viable therapeutic option to treat inflammatory diseases and improve tumor therapy.

## 1. Introduction

**Figure 1:**
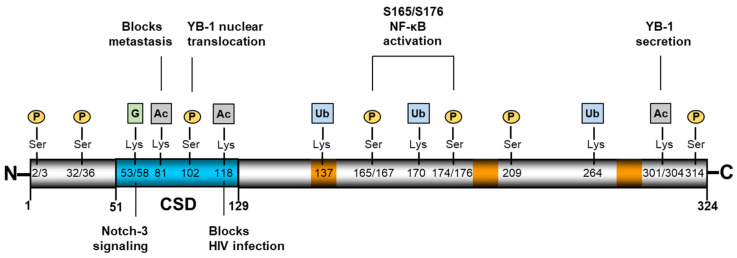
Structural organization of YB-1. YB-1 is a 324 amino-acid protein comprising an alanine/proline-rich N-terminus, a highly conserved nucleic acid binding cold-shock domain (CSD, blue), and a large, disordered C-terminus [5]. YB-1 contains three nuclear localization signals (orange), which comprise stretches of positively charged amino acids. Numerous post-translational modifications have been identified, several of which are shown here along with the known activities. Figure 1 residues, modification types, and corresponding references are shown in Table A1.

Y-box binding protein-1 (YB-1, YBX1) belongs to the evolutionarily conserved family of cold-shock domain proteins, which engage in DNA and RNA binding activity [1,2]. In eukaryotes, the protein is composed of an alanine/proline-rich N terminus, a central cold-shock domain (CSD) that binds DNA and RNA, and a C-terminal charged zipper characterized by alternating stretches of positively and negatively charged amino acids (Figure 1) (for more information on the structure of the CSD and its nucleic acid binding properties, see [2]). Thus, YB-1 functions as a nucleic acid chaperone and, via its interaction with numerous proteins, exerts pleiotropic functions in cellular processes such as proliferation, differentiation, DNA damage repair, RNA splicing, and cellular stress responses [3,4]. 

In healthy tissue, YB-1 is primarily cytoplasmic, where it plays an important role in regulating various aspects of RNA biology [6]. Together with polyA-binding protein (PABP), YB-1 is a major constituent of the cytosolic mRNA-containing ribonucleoprotein (mRNP) complexes. A high YB-1:mRNA ratio represses translation, whereas low levels of YB-1 facilitate the translation of certain genes. Examples of YB-1-regulated genes include the epithelial-to-mesenchymal transition factors SNAIL, TWIST, and ZEB2, as well as those contributing to metastasis, i.e., NRF2, HIF1α, and G3BP1 [7,8]. The ability of multiple YB-1 molecules to bind a single mRNA to form filament-like structures appears to be regulated in part by charged residues within the C-terminal domain [9,10]. This domain also appears responsible for the binding of poly(ADP-ribose) (PAR) [11]. Phosphorylation of YB-1 also appears to play a role in regulating its nucleic acid binding activities, as well as its subcellular localization [12,13,14] (for a more detailed discussion on YB-1 RNA/DNA binding, see [3,14]). Upon activation, YB-1 translocates to the nucleus, where it can influence the transcription of genes involved in cell division, immune response, apoptosis, multidrug resistance, and tumor growth [12]. YB-1 stimulates poly(ADP-ribose) polymerase 1 (PARP1) activity, thereby influencing DNA damage repair, which also results in the PARylation of YB-1 [11]. YB-1 is upregulated in tumors and its nuclear localization is associated with a more aggressive phenotype indicating a poor prognosis [15,16,17]. Whether YB-1 exerts its influence solely via RNA regulation or also via ssDNA is still being debated [18]. Furthermore, YB-1 is secreted from cells and is highly abundant in serum. The acetylation and ubiquitination of YB-1 play a central role in regulating protein secretion, as well as intracellular stability [19,20]. Notably, four independent studies employed genome-wide proteomic profiling to examine ubiquitination and identified several sites on YB-1 [21,22,23,24]. One of these studies found that retinoblastoma binding protein 6 (RBBP6), an E3 ubiquitin ligase, interacts with YB-1, resulting in its ubiquitination and proteasomal degradation [25]. Recently, Breitkopf et al. identified a specific guanidinylation of YB-1 at residues K53 and K58, which lie within the highly conserved cold-shock domain [26]. This modification was observed in sera from lupus-prone mice and patients with SLE, correlating with disease activity, especially in patients with lupus nephritis. Extracellular YB-1 acts as a ligand for membrane receptors, such as Notch3 and TNFR1, thereby activating intracellular signaling cascades [27,28]. Thus, YB-1 is a key player in orchestrating inflammatory processes and immune cell phenotypes. Inflammation and cancer are known to share common pathomechanisms, such as the recruitment of infiltrating macrophages and development of an inflammatory microenvironment. Here, since YB-1 is linked to both fields, we will summarize and combine recent results to provide new insights.

In serum samples from cancer patients, a fragment of YB-1/p18 has been detected with a high prevalence in a variety of cancers [29,30]. These findings hint at a prominent role for secreted YB-1 and its metabolites. However, it has not been established whether YB-1 is cleaved intracellularly and p18 secreted, or if YB-1 is secreted and then cleaved to p18 in serum.

## 2. Extracellular YB-1

YB-1 is secreted via a non-classical secretion pathway in diverse cell types, e.g., by granulocytes, lymphocytes, monocytes, renal, and hepatic cells, in response to inflammatory stimuli such as PDGF, TGF-β, and LPS [31,32]. Post-translational modifications of YB-1, such as acetylation and ubiquitination, have been shown to regulate its secretion [20,31]. Extracellular YB-1 engages in chemoattractant activity [32,33] and displays pro-mitogenic effects, exhibiting involvement in tumor progression [31]. In contrast, a recent study showed that extracellular YB-1 from HEK293T cells significantly inhibited the proliferation of receiving cells, and that such inhibition was associated with a G2/M cell cycle arrest [34]. Importantly, the observed effect was not cell specific.

Extracellular YB-1 functions as a non-canonical ligand for the Notch3 receptor and thereby modulates Notch3 signaling [28,35]. Although Notch3 appears to play an important role in tumorigenesis and chemotherapy resistance, the molecular mechanisms by which it exerts these effects are less clear [36]. In human kidney diseases, Notch3 has been shown to be upregulated and to modulate inflammation and fibrosis during kidney injury [37]. Secreted YB-1 also contributes to monocyte/macrophage recruitment and differentiation after LPS-stimulation in vitro and, in a mouse model of kidney inflammation in vivo, supports the kidney’s immunomodulatory function [32,38]. The recent study by Hessman et al. identified a novel role of extracellular YB-1, which, together with progranulin, interferes with the binding of tumor necrosis factor (TNF) to its receptor TNFR1 [27]. Oligomerization of the TNF receptor is necessary to induce signaling. This requires both dimerization of the receptor chains, via the pre-ligand assembly domain, and ligand binding [39]. Thus, it may be that YB-1/progranulin interferes with oligomer formation, thereby preventing signal transduction, although whether this occurs in an autocrine or paracrine manner is unclear. Here, a more detailed analysis of the secretion kinetics is required. One can envision that a delayed release of YB-1/PGRN with respect to TNF could be an effective means to terminate TNFR signaling. In addition to the ability of YB-1 to influence the binding of TNF to its receptor extracellularly, the signals transduced by TNFR1 have also been shown to be altered in the absence of YB-1 intracellularly [40].

## 3. TNF Receptor-1 (TNFR1) Signaling Pathway 

TNF is produced as a 26 kDa transmembrane protein (mTNF). It is expressed on the cell surface, where it is actively cleaved, via the TNF-converting enzyme (TACE), to produce a 17 kDa soluble (sTNF) homotrimer, which is then released and is detectable in blood plasma [41]. The secreted form of this molecule is a potent inflammatory cytokine. TNF is involved in a variety of cellular processes, such as inflammation, cell proliferation, differentiation, and the induction of apoptosis. It is known to play a critical role in the pathogenesis of chronic inflammatory diseases and cancer [42,43,44]. TNF exerts cellular activities by its two receptors: TNFR1 is expressed in all human tissues, whereas TNFR2 is expressed mainly in immune cells, neurons, and endothelial cells [45,46]. Both receptors share a similar structure in their N-terminal extracellular domain (ECD), which is composed of four cysteine-rich domains (CRDs), and a single α-helical transmembrane domain [47]. However, they differ in the cytoplasmic domain, where TNFR1 possesses a death domain (DD) and TNFR2 does not [48]. Death domains are common to all death receptors, being found in Fas/Apo-1/CD95, TNFR1, TRAIL-R2, DR6, p75-nerve growth factor receptor (NGFR), and ectodermal dysplasia receptor (EDAR), which all belong to the TNFR superfamily and can induce apoptosis. Signaling via TNFR1 initiates pro-inflammatory responses and induces cell death, but it can also promote cell survival [49].

**Figure 2:**
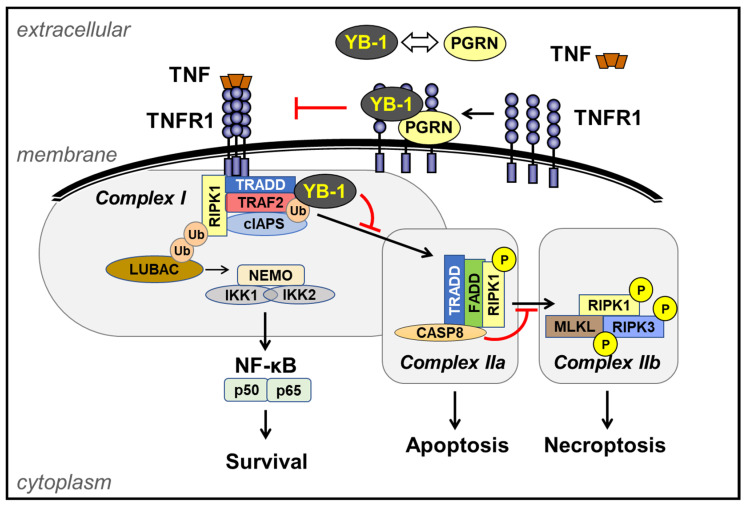
Relationships of different TNFR1 complexes. Upon the binding of TNF to TNFR1, TNFR1-associated death domain (TRADD) is recruited, which then binds to receptor-interacting protein kinase 1 (RIPK1), TNFR-associated factor 2 (TRAF2), and the cellular inhibitor of apoptosis protein 1 and 2 (cIAP1/2) to form complex I [50,51]. cIAP1/2 and the linear ubiquitin chain assembly complex (LUBAC) add Met1- and K63-linked polyubiquitin (Ub) chains to RIPK1. This linkage-specific ubiquitination stabilizes RIPK1, amplifying its signal. K63-linked Ub chains on RIPK1 recruit the transforming growth factor-β (TGFβ)-activated kinase 1 (TAK1) complex, comprising TGFβ-activated kinase 1 and mitogen-activated protein kinase (MAPK)-binding proteins 2 and 3 (TAB2 and 3) and TAK1. The TAK1 complex phosphorylates the IκB kinase (IKK) complex. This results in the translocation of NF-κB into the nucleus, leading to gene transcription [57,58,59]. RIPK1 is deubiquitinated by cylindromatosis tumor suppressor protein (CYLD), facilitating its dissociation from complex I. Recruitment of Fas-associated protein with death domain (FADD), and caspase 8, results in the formation of complex IIa which triggers apoptosis; active caspase 8 also cleaves RIPK1, preventing it from forming complex IIb [60,61,62,63]. In the absence of caspase 8 protease activity, RIPK3 and MLKL (mixed lineage kinase domain-like pseudokinase) are recruited to form complex IIb and together trigger necroptosis. Extracellular YB-1, together with progranulin (PGRN), competes with TNF for binding to TNFR1 [27].

Upon TNF-induced receptor oligomerization, signaling proteins such as the TNF receptor-associated death domain protein (TRADD) and receptor-interacting serine/threonine-protein kinase 1 (RIPK1), and TNF receptor-associated factor 2 (TRAF2) are recruited to the membrane forming complex I (Figure 2). Here, TRAF2, an E3 ubiquitin ligase, plays an important role in the activation of NF-κB [50,51], by recruiting the cellular inhibitor of the apoptosis protein (cIAP1/2). Together, cIAP1 and 2 attach K11-, K48- and K63-linked polyubiquitin to RIPK1 and to themselves [52,53]. This enables recruitment of the linear ubiquitin assembly complex [54,55] and a complex consisting of TAK1-binding protein 1 and 2/3 (TAB1-TAB2/3)-transforming growth factor β-activating kinase 1 (TAK1), referred to as a TAB/TAK complex. The binding of both complexes in turn allows for the subsequent recruitment and activation of the inhibitor of κB (IκB) kinase, IKKα/β, through the NF-κB essential modulator (NEMO), forming the IKK complex [56]. This results in the activation of canonical NF-κB signaling and the mitogen-activated protein kinases (MAPKs), leading to the upregulation of a plethora of pro-survival and inflammatory genes (Figure 2) [56].

For most cells, the binding of TNF to TNFR1 does not induce death, due to the presence of two sequential checkpoints, distinguished as early and late. The early checkpoint is transcription-independent and initiated by RIPK1 ubiquitination. The modification maintains RIPK1 in a survival mode and prevents it from activating downstream death-signaling molecules. Disruption of this checkpoint switches RIPK1 into a death-inducing molecule, which upon TNFR1 ligation is able to induce apoptosis or necroptosis. The late checkpoint requires the transcription of pro-survival genes by NF-κB. These gene products inhibit the death-signaling machinery. If the late checkpoint is disrupted, e.g., by inhibiting transcription, cells undergo apoptosis in response to TNFR1 ligation [49]. 

In a recent study from our group, the role of YB-1 in TNFR1 signaling by regulating NF-κB activation in monocytes/macrophages and cancer cell lines has been described [40]. We identified a central role for YB-1 in TNFR1-mediated cell fate signaling. The loss of YB-1 destabilizes TRAF2, thereby abrogating NF-κB activation and promoting apoptosis. Identifying the molecular mechanism of YB-1-mediated NF-κB activation may reveal therapeutic targets for interventions in cancer.

Based upon our current understanding of inflammatory diseases, novel treatment strategies have been developed that target cytokines or interfere with signaling cascades. Popular approaches include cytokine-blocking antibodies (Infliximab: TNF-α), soluble TNF receptors (Onercept/Etanercept) and the IL-1β receptor (Anakinara) [64,65]. It has been observed that rheumatoid arthritis patients treated with anti-TNF therapy showed improved clinical symptoms, such as reduced joint damage and bone destruction. Similarly, rheumatoid arthritis patients who also suffer from chronic kidney disease showed improved kidney function [66]. Strong evidence now exists for the role of macrophage-derived TNF in the progression of atherosclerosis [67,68,69,70]. Despite anti-TNF therapies having well-established efficacy, the enhanced risk of lymphomas or recurrence of latent infection remains a major disadvantage [65,71,72,73]. Therefore, drug development has focused on interference with cytokine receptors, thereby blocking the activation of signaling pathways to prevent the formation of an inflammatory milieu. In inflammatory models, TNF-mediated NF-κB activation upregulates adhesion molecules that are required for the extravasation of monocytes [74]. Recently, it has been shown that mast cell-derived TNF directly primes the circulating neutrophils by TNFR1, and is also vital for endothelial cell activation [75]. The modulation of tumor necrosis factor receptor by YB-1 may be relevant to numerous diseases, including diabetic nephropathy, systematic lupus erythematosus, liver fibrosis, and infectious and cancerous diseases in which TNF plays a major role in the disease pathology, e.g., by establishing an inflammatory milieu and recruiting tumor-associated macrophages (TAMs).

## 4. Targeting YB-1 Influences Cell Death Decisions

Apoptosis is a form of programmed cell death that is initiated by caspases. This protease family is subdivided into initiator caspases (e.g., caspase 8, 10) and executioner caspases (e.g., caspase 3, 7) [76]. There are two known pathways that lead to apoptosis: the intrinsic pathway and the extrinsic pathway. The intrinsic pathway begins when injury within the cell results in cytochrome C release from mitochondria leading to apoptosome activation. The extrinsic apoptotic pathway is triggered by the binding of a ligand to its cell death-inducing surface receptors, e.g., FasL to Fas/Apo-1/CD95 or TNF to TNFR1. In both the intrinsic and extrinsic pathway of apoptosis, signaling results in the activation of caspases that act in a proteolytic cascade [77]. PARylation has also been implicated in inducing necroptotic cell death, due to NAD depletion, parthanatos, and autophagic cell death [78]. Given the ability of YB-1 to stimulate PARP1 activity and to be PARylated [11], one could envision that YB-1 exerts an influence on these pathways as well. However, to our knowledge, this connection has not yet been investigated.

### 4.1. Myeloproliferative Neoplasia (MPN) 

JAK2 is required for stem cell maintenance and the mutations in JAK2 that occur with aging pose a risk for the development of MPN. JAK2 inhibitors reduce the proliferation, although cancer still persists. To gain insight into the underlying mechanism, Jayavelu et al. identified ~22,000 phosphorylation sites in ~4000 proteins, of which ~5200 sites (on ~1800 proteins) were JAK2 mutant (V617F)-specific [79]. The top 15 candidates, including YB-1, were selected for an siRNA screening. Interestingly, only YB-1 suppression sensitized JAK2VF cells to JAK2 inhibition. To investigate the mechanism, they then analyzed specific changes in YB-1 phosphorylation using diverse kinase inhibitors. It was not surprising that S165 and S176 were among the sites listed, as these are responsible for NF-κB activation [80,81]. Here, the authors identified S30 and S34 as phospho-sites that decreased upon MEK/ERK inhibition. YB-1 was found to be interacting with ribonucleoproteins, mRNA splicing factors, and ribosomal proteins. To identify the targets of YB-1-mediated splicing, they performed RNA-seq analysis. The major pathways affected were ERK signaling and programmed cell death. Given the role of YB-1 in mRNA splicing, transcriptome profiling of murine JAK2VF cells was performed, and led to a 70% increase in intron retention (unspliced) compared to controls. Phosphoproteome analysis showed many of the phospho-sites reduced in YB-1-deficient JAK2VF cells are ERK substrates, including MKNK1 and MCL1. Furthermore, the addition of a JAK inhibitor ablated ERK signaling. Treatment with mRNA splicing inhibitors confirmed that MKNK1 expression is dependent on efficient mRNA splicing, which is mediated by YB-1. Thus, Jayavelu et al. clearly demonstrated that YB-1 is required to maintain ERK signaling downstream of JAK2VF. Importantly, the genetic inactivation of YB-1 did not perturb hematopoiesis. However, combining pharmacological JAK inhibition with YB-1 inactivation leads to apoptosis in JAK2-dependent mouse and primary human cells [79]. Thus, targeting YB-1-induced ERK signaling in combination with JAK2 inhibitors is an effective therapeutic strategy to eradicate cells harboring JAK2 mutations.

### 4.2. Chronic Myeloid Leukemia

YB-1 regulates m6A-tagged mRNA stability by interacting with IGF2BPs. The study by Feng et al. provides evidence that m6A modification is required for the maintenance of *BCL2* mRNA stability, which is necessary for maintaining the survival of myeloid leukemia cells. The authors established a link between YB-1 and Bcl-2 expression. Bcl-2 is known to be upregulated in AML and leukemia stem cells [82,83]. Inhibition of Bcl-2 by a selective mimetic has proven to be an efficient strategy in eradicating leukemic stem cells in the clinic [84]. It has also revealed a novel mechanism for how YB-1 selectively functions in regulating the survival of myeloid leukemia cells and indicates a potential therapeutic approach for treating myeloid leukemia, as well as other cancers [85,86,87].

### 4.3. Systemic Lupus Erythematosus (SLE)

In late 2020, YB-1 was shown to be responsible for the disturbed homeostasis of activated T cells in SLE. YB-1 interacts with p53, and they regulate each other’s function [88]. In the absence of YB-1, free p53 upregulates the expression of pro-apoptotic proteins, such as NOXA and PUMA, thereby activating the intrinsic apoptosis pathway [89,90]. Conversely, the overexpression of YB-1 markedly enhances survival in activated T cells. YB-1 appears to support the expression of the anti-apoptotic proteins Bcl-2 and Bcl-xl [91]. Meltendorf et al. showed that YB-1 also induces the expression of Akt, which is a survival factor in CD4^+^ T cells [92]. In this line, knockdown of YB-1 was found to reduce Akt expression, which suggests this protein–protein interaction has a stabilizing effect [13,93]. Akt1 overexpression has been shown to partially rescue SLE T cells from apoptosis. PI3K inhibition enhances apoptosis in control cells, suggesting this pathway is involved in regulating CD4^+^ T cell survival. YB-1 expression in CD4^+^ T cells has also been shown to correlate strongly with survival and cytokine production [90]. Acetylation of YB-1 has also been shown to block the replication of HIV in CD4^+^ T cells [94].

### 4.4. T Cell Leukemia

YB-1 is a central player in driving the proliferation of primary and malignant T cells. Within activated T cells, the extrinsic apoptotic pathways regulate homeostasis. One pathway is activation-induced cell death (AICD), which is triggered by the binding of FasL to its death-inducing receptor Fas/Apo-1/CD95, or by the binding of TNF to TNFR1. Since both ligands and receptors are upregulated during T cell activation, they can counteract excess clonal T cell expansion and restore homeostasis. Patients whose AICD pathway is defective suffer from autoimmune lymphoproliferative syndrome (ALPS) [95]. Stimulation of primary CD4^+^ T cells has been shown to induce YB-1 expression. This observation is similar to the stimulation-dependent YB-1 enhancement seen in non-lymphoid cells [96,97,98,99]. Low constitutive YB-1 levels may be required for T cell homeostasis [100,101]. Co-stimulation with CD3/CD28 significantly induces YB-1 protein expression, compared to stimulation with CD3 alone. This suggests that YB-1 mediates the proliferative effects of CD28, such as IL-2 mRNA stabilization and expression [102], which are required for clonal T cell expansion [89].

### 4.5. Ovarian Cancer 

Seeking to improve the potency of a lead compound as a treatment for ovarian cancer, Tailor et al. [54] used bioisosterism to generate SU056, which induces cell cycle arrest and apoptosis in cancer cell lines, and demonstrates an improved ability to inhibit tumor growth in mice. A cellular thermal shift assay identified six proteins as targets of SU056, including YB-1. Given the prominent role of YB-1 in cancer, the authors focused on this candidate, showing that SU056 binds to YB-1, thereby resulting in an enhanced proteolytic degradation of the YB-1 protein. The loss of YB-1 results in a subsequent reduction in the expression of YB-1 target genes, including multidrug resistance 1 (MDR1) and multiple components of the spliceosome. In contrast to other studies, an upregulation of the pro-apoptotic protein Bax was observed, with no loss of Bcl2. Regardless, the end effect is that SU056 treatment sensitizes tumors to chemotherapy. It will be interesting to see how effective this compound is against myeloproliferative neoplasia and leukemia.

## 5. Outlook 

To date, there are no established therapies in clinical practice that specifically target YB-1 [79]. However, the genetic inactivation of YB-1 does not appear to perturb steady-state hematopoiesis and no functional impairment of hematopoietic stem and progenitor cells has been observed. Thus, YB-1 appears to be a safe target. In cancer, higher YB-1 expression and nuclear localization have been linked its ability to induce multi-drug resistance and to a poor prognosis [88,103,104]. Thus, drugs enforcing a cytosolic localization of YB-1 should be more effective at preventing YB-1’s pro-survival signaling. Several inhibitors have been reported that influence the subcellular localization of YB-1 [105]. These compounds belong to two categories: (1) molecules that induce nuclear translocation, e.g., HSc025, which disrupts YB-1’s binding to poly(A)-binding protein (PABP), thereby disrupting its cytosolic retention; and (2) molecules that promote cytoplasmic retention, e.g., fisetin, which binds to the cold-shock domain and prevents the phosphorylation of Ser102, which precludes nuclear translocation [89,106,107]. Recently, theaflavin-3-gallate (TF2A), derived from black tea, has been shown to improve the stability of YB-1. This study suggests that YB-1 might be a potential molecular target modulating hypothalamic neural stem/progenitor cell (htNSC) senescence [108]. Of note, determining the molecular mechanism underlying the YB-1-mediated NF-κB activation we described for TNFR1 signaling should aid in identifying new therapeutic targets for intervention in cancer. We predict that targeting YB-1 should sensitize cells to TNF-induced apoptosis, a potent strategy to attack cancer cells [4,88,109].

The recent findings from several studies provide novel insights into YB-1’s functions, but also raise new questions. Concerning extracellular YB-1, how many post-translational modifications does it possess, which enzymes are responsible, and how do these modifications affect protein stability and receptor affinity? Could differences in post-translational modifications be responsible for the differences in the observed activities of extracellular YB-1 that have been reported? Do these modifications contribute to the development of autoantibodies against YB-1 [110,111,112], and what is the relationship between the presence of autoantibodies in serum and disease progression?

Numerous post-translational modifications are also reported for intracellular YB-1. Are specific modifications associated with distinct activities or subcellular compartments of YB-1? Here, extensive work is required to assign functions to the known modifications, and to define the mechanisms by which the known modifications exert their function. Do these modifications function independently of one another, or are they part of larger, as yet uncharted, regulatory networks? To date, only a handful of receptors are known that utilize YB-1 as a signaling component. Whether or not YB-1’s role in signal transduction is independent of its mRNA splicing and translational regulatory activities is yet to be demonstrated, but clearly each of these activities can impact upon the life and death decisions of the cell.

## 6. Conclusions

From the discovery of new post-translational modifications and activities, the recent advances in the literature have clearly shown us one thing: we still have a lot to learn about YB-1! From the concept of “one protein: one function”, YB-1 has stepped outside the box and pushed the limits of our creativity, forcing us to design novel concepts to explain the multiplicity of functions that one protein can fulfill. We must keep this in mind when interpreting the results of studies in which YB-1 expression has been manipulated using shRNA or similar strategies. How does the absence of cold-shock proteins affect the expression of other proteins? Whose expression is now induced or inhibited, whose splicing is altered, and if so, is the function of that protein also altered? Take MCL1, for example: the long isoform is anti-apoptotic, whereas its shorter isoforms are pro-apoptotic. Thus, it appears that only through the use of RNA sequencing, combined with -omics technologies, will we be able to gain a complete view of the changes occurring within these model cell systems. Certainly, the application of inducible knockout mouse models will also help us to gain a broader view, particularly when it comes to cell–cell interactions and inter-organ crosstalk.

In the field of cancer research, the results delivered in recent studies are quite encouraging. YB-1 appears to be an ideal target which, in combination therapy, may break the proliferative cycle of malignancies. Clearly, there is now a great need to identify specific inhibitors that selectively target YB-1 or, alternatively, to find combination therapies, such as ruxolitinib and trametinib (combined JAK and ERK inhibition), that target activities mediated by YB-1 [79]. The translation of these studies into the clinic and their application to other cancer models will soon reveal the efficacy of these strategies. Have we finally found the “magic bullet” that so many previous reports have promised? Time will tell.

## Appendix A

**Table A1:** The residue, type of modification, and corresponding references to Figure 1.

Site on YB-1	Type of PTM	Ref.
S32	Phosphorylation	[79]
S36	Phosphorylation	[79]
K53	Guanidinylation	[26]
K58	Guanidinylation	[26]
K81	Acetylation	[7]
S102	Phosphorylation	[93]
K118	Ubiquitination	[81]
K137	Ubiquitination	[24]
K170	Ubiquitination	[24]
S165	Phosphorylation	[81]
S167	Phosphorylation	[24]
S174	Phosphorylation	[24]
S176	Phosphorylation	[80]
S209	Phosphorylation	[24]
K264	Ubiquitination	[24]
K301	Acetylation	[31]
K304	Acetylation	[31]
S314	Phosphorylation	[79]
